# Application of risk factors for venous thromboembolism in patients with multiple myeloma starting chemotherapy, a real‐world evaluation

**DOI:** 10.1002/cam4.1927

**Published:** 2018-12-25

**Authors:** Hailey A. Baker, Alexandra R. Brown, Jonathan D. Mahnken, Theresa I. Shireman, Carol E. Webb, Brea C. Lipe

**Affiliations:** ^1^ University of Kansas School of Medicine Wichita Kansas; ^2^ University of Kansas Medical Center Kansas City Kansas; ^3^ Brown University School of Public Health Providence Rhode Island; ^4^ University of Kansas Cancer Center Kansas City Kansas; ^5^ University of Rochester Medical Center Rochester New York

**Keywords:** guidelines, multiple myeloma, risk, thromboprophylaxis, venous thromboembolism

## Abstract

**Introduction:**

Within the first year of diagnosis, up to 1 in 3 multiple myeloma (MM) patients will experience a venous thromboembolism (VTE). The International Myeloma Working Group (IMWG) has thromboprophylaxis guidelines that stratify patients into low or high risk for thrombosis and subsequently recommend thromboprophylaxis, but it is unknown if these recommendations are being followed or if they are effective. The purpose of this study was to assess efficacy of the IMWG guidelines and investigate other potential VTE risk factors.

**Methods:**

Study participants were treated at the University of Kansas Medical Center between 2007 and 2013, and charts were reviewed to extract data. Cases (MM and VTE) were matched to controls (MM and no VTE) at approximately 1:3 ratio based on gender, age (±5 years), and time of MM diagnosis (±5 years).

**Results:**

A total of 80 cases and 211 controls were matched. Most patients (82%) were considered high risk for experiencing a VTE at the time of their MM diagnosis and 18% were considered low risk. Neither risk category (*P* = 0.16) nor thromboprophylaxis at baseline (*P* = 0.37) predicted VTE, though cases were more likely than controls to have an increased risk of thrombosis at the time of clot compared to their baseline risk (*P* = 0.09).

**Conclusion:**

Our results suggest that IMWG guidelines are not being consistently followed and therefore could not be validated. Additional risk factors were not identified, but risk for VTE may change over time suggesting patients may require ongoing assessment of VTE risk and thromboprophylaxis throughout the disease course.

## 1 INTRODUCTION

Multiple myeloma (MM) is an incurable malignancy of plasma cells affecting approximately 30 000 new patients in the United States in 2016 that also portends an increased risk of venous thromboembolism (VTE).^1^, ^2^, ^3^ The mechanism of increased risk of VTE in MM is likely multifactorial and includes pathophysiologic changes, patient‐specific factors, and treatment‐related factors.^4^ Direct paraprotein interactions, altered fibrin clot formation, and upregulation of inflammatory cytokines have all been implicated in the pathophysiology of VTE in patients with plasma cell dyscrasias.^5^, ^6^, ^7^ Multiple myeloma generally affects patients over 60 years old, so patients commonly have comorbidities that increase their risk for VTE.^8^, ^9^ Treatments used for MM can further increase the risk of VTE. Immunomodulators combined with high‐dose dexamethasone or anthracyclines are the most thrombogenic with VTE rates of 14%‐75%.^10^

Venous thromboembolisms carry significant clinical implications on morbidity^11^, ^12^ and mortality in cancer patients,^13^ with a decreased survival in multiple myeloma specifically.^14^ Because cancer patients with an initial VTE are at increased risk of a second VTE, they are recommended for prolonged or indefinite anticoagulation after an initial event.^15^ However, anticoagulation is not without risk. There is a 2‐fold increased risk of major bleeding events seen in cancer patients on anticoagulants.^16^ Given the high risk of VTEs in MM and the potential complications of such an event, reducing the risk of VTE for patients with MM remains of paramount importance.

In an attempt to reduce the rates of VTE for patients with MM, the International Myeloma Working Group (IMWG) created guidelines for thromboprophylaxis. These guidelines attempt to identify groups of patients at highest risk for VTE and recommend different thromboprophylaxis strategies to prevent VTE.^10^ The IMWG recommendations regarding risk factors for VTE (Table 1) have been extrapolated from a variety of different studies conducted on different patient populations, including unselected patients and patients with general malignancy.^13^, ^17^, ^18^ In MM specifically, there is a well‐established risk of VTE with therapy,^5^ but attempts to identify additional risk factors for thrombosis have been inconclusive.^19^, ^20^, ^21^, ^22^ Thromboprophylaxis recommendations within the IMWG guidelines are therefore based on limited data. Comparative effectiveness studies of aspirin 100 mg/d vs LMWH^19^ and aspirin 100 mg/d, LMWH, or a fixed dose of warfarin at 1.25 mg/d^23^ found no significant difference in VTE rates. These trials of VTE prophylaxis may have limited real‐world applicability as both studies were done as substudies of therapeutic trials that exclude patients with many of the risk factors associated with VTE. The efficacy of the IMWG guidelines have not been tested directly in MM patients, and the compliance with these guidelines for patients on therapy is likewise unknown. Given the potential consequences of the recommendations regarding efficacy, cost, and patient comfort for oral vs injectable thromboprophylaxis, we sought to validate the IMWG recommendations and evaluate other potential risk factors for VTE among patients with MM treated at the University of Kansas Medical Center (KUMC).

**Table 1:** Categories of study variables collected

IMWG risk factors	Additional risk factors	Disease information	Medications
Obesity (>30 kg/m^2^) Renal disease (GFR < 60) Trauma, surgery, or infection requiring hospitalization Prior VTE Diabetes Central venous catheter Pacemaker Known clotting disorder Cardiac disease^a^ Immobilization	Hypertension requiring treatment Smoking status History of bleeding requiring a blood transfusion Family history of thrombosis Karnofsky < 70% Other malignancy	Disease stage M‐spike at diagnosis Age at diagnosis Free light chain ratio at diagnosis	Aspirin LMWH Warfarin Erythropoietin stimulating agents Dexamethasone Progesterone Chemotherapy

a Cardiac disease defined as NYHA failure >1, prior MI, or revascularization

## 2 METHODS

### 2.1 Study design

We utilized a case‐control design by matching cases (m) to controls (n) with a goal of approximately one case: three controls where possible. Matching occurred based on gender, age (±5 years), and date of diagnosis (±5 years). A retrospective chart review was performed by two trained investigators to confirm the date of diagnosis of MM for identified patients and to extract study variables.

### 2.2 Setting

Patients with MM who were treated at KUMC, an academic tertiary medical center, between 2007 and 2013 were included in the study. This study was approved by KUMC's internal review board.

### 2.3 Participants

Only patients with at least 1 year of treatment data were included in the study. Patients were identified using the Healthcare Enterprise Repository for Ontological Narration (HERON) database using ICD‐9 diagnosis codes (203, 203.1, 238.6, 415, and 452) and KUMC's cancer registry. HERON is a research tool that conducts cohort discovery queries of the electronic medical records.^24^, ^25^ Data from HERON were confirmed by chart review of each case and control. Cases were defined as patients who experienced a VTE after their MM diagnosis. Venous thromboembolism was defined as any VTE of the upper or lower extremity or pulmonary embolism, excluding superficial venous thrombosis. Documented evidence of a clot was confirmed on chart review by ultrasound results or physician documentation in chart. Controls were defined as patients with MM who had not experienced a VTE after their MM diagnosis.

### 2.4 Variables

Demographic variables included gender, race, and date of death (where applicable). All additional variables collected on cases and controls are outlined in Table 1. The outcome of interest was whether or not VTE prophylactic therapy was concordant with IMWG guidelines based upon patient risk factors and thromboprophylaxis used. IMWG guidelines were considered as “followed” for low‐risk patients if the patient was low risk and taking ASA. The guidelines were considered “followed” for high‐risk patients if the patient was taking LMWH or warfarin. IMWG guidelines were considered not followed if either of these conditions were not met.

### 2.5 Data sources/measurement

Chart‐abstracted data were entered into standardized data forms in the Research Electronic Data Capture (REDCap) database^26^ on a KUMC secured server. Data for controls were collected at the time of diagnosis ±30 days. Data for cases were collected at the time of diagnosis ±30 days and at the time of VTE ±30 days.

### 2.6 Bias

Our best efforts were made to reduce bias by using a matched design to eliminate confounding variables of age, gender, and date of diagnosis.

### 2.7 Quantitative variables

Patient risk categories were defined in concordance with IMWG guidelines as high or low risk based on the presence of one or more IMWG risk factors (Table 1). If zero or one IMWG risk factor was present, the patient was considered low risk. Patients not on chemotherapy were considered low risk. If more than one risk factor was present, the patient was considered high risk.

### 2.8 Statistical methods

A matched analysis was conducted because of our study design. To assess whether the rate of warfarin and LMWH use differed among low‐ vs high‐risk patients, we used a Cochran‐Mantel‐Haenszel (CMH) test to test for association between risk category and warfarin and LMWH use, controlling for this matched design. Following this test, we descriptively estimated the proportion of subjects that received warfarin or LMWH among the both high and low risk and calculated a 95% CI for these proportions. Using conditional logistic regression, we examined an association between VTE events, medications use, and the interaction of medication use with risk category for the matched patients at the time of diagnosis and at the time of the VTE. All analyses were performed using SAS version 9.4M1. Statistical significance was defined as *α* < 0.05. To determine whether additional risk factors or combinations of risk factors were correlated with an increased risk of VTE, we conducted an exploratory analysis and therefore did not adjust our level of significance for multiple testing. Patients with missing data were excluded from analysis.

## 3 RESULTS

There were a total of 306 patients with multiple myeloma identified from 2007 to 2013. Fifteen patients were excluded from the cohort due to missing data, nine of which were excluded due to missing diagnosis date. No significant difference in overall survival was observed between those included and excluded (*P* = 0.35). Our final cohort consisted of 80 cases with a treatment‐associated VTE (15.7% of all patients) and 211 matched controls for a final cohort of 291 participants. Among 80 cases, we identified four controls for one case, three controls for 57 cases, two controls for 14 cases, and one control for eight cases. The mean time difference between matched controls and cases is 0.36 years (SD 1.4 years). Baseline patient demographics for cases and controls are presented in Table 2. Our cases and controls were well matched without significant differences being detected across age, gender, or race. The only significant differences between cases and controls were for performance status (Karnofsky or ECOG score). Controls had larger proportions of patients with better performance status (72.51% controls vs 53.75% cases, *P* = 0.01). No significant differences between VTE risk factors were observed between cases and controls. Furthermore, no significant differences in IMWG risk factors for VTE found between high‐risk cases and high‐risk controls.

**Table 2:** Patient characteristics at baseline

Variables	Total (n = 291)	Cases (n = 80)	Controls (n = 211)	*P*‐value
Age (years)	62.26	61.00	63.00	0.56
Median [Q1, Q3]	[35.00, 84.00]	[35.00, 81.00]	[36.00, 84.00]	
Gender, N (%)				
Males	137 (47.08)	37 (46.84)	100 (47.39)	0.93
Females	153 (52.58)	42 (53.16)	111 (52.61)	
Time from diagnosis to data collection (years) [Median: Q1, Q3]	5.40 [2.06, 18.72]	5.41 [2.06, 17.34]	5.40 [2.15, 18.72]	0.97
Race, N (%)				
Caucasian	183 (62.89)	47 (60.26)	136 (64.76)	0.88
African American	33 (11.34)	11 (14.10)	22 (10.48)	
Asian	1 (0.00)	0 (0.00)	1 (0.00)	
Other or unknown	70 (24.05)	20 (25.00)	50 (23.70)	
Deceased as of 05/2016, N (%)	85 (29.2)	27 (33.75)	58 (27.49)	0.32
Median time to death from diagnosis (years) [Median: Q1, Q3]	5.30 [4.79, 5.98]	5.58 [4.98, 6.79]	5.01 [4.43, 6.06]	0.16
ISS disease Stage, N (%)				
Stage I	33 (11.34)	9 (12.00)	24 (11.88)	0.67
Stage II	40 (13.75)	14 (18.67)	26 (12.87)	
Stage III	66 (22.68)	17 (22.67)	49 (24.26)	
Unknown	138 (47.42)	35 (46.67)	103 (50.99)	
High‐risk cytogenetics, N (%)^a^	72 (24.7)	26 (32.50)	46 (22.44)	0.15
Number of IMWG risk factors for VTE, N (%)				
None	21 (7.22)	4 (5.00)	17 (8.06)	0.23
1	32 (11.00)	7 (8.75)	25 (11.85)	
>1	238 (81.79)	69 (86.25)	169 (80.09)	
Mean (SD)	1.54 (1.22)	1.61 (1.34)	1.40 (1.13)	
Type of induction therapy, N (%)				
Steroid only	12 (4.12)	2 (2.50)	10 (4.74)	0.68
RVd	57 (19.89)	18 (22.50)	39 (18.48)	
CyBord	7 (2.41)	0 (0.00)	7 (3.32)	
Rd	47 (16.15)	14 (17.50)	33 (15.64)	
Vd	72 (24.74)	19 (23.75)	53 (25.12)	
Other	84 (28.87)	25 (31.25)	59 (27.96)	
None	8 (2.75)	1 (1.25)	7 (3.32)	
Karnofsky < 70 or ECOG > 1, N (%)				
Yes	33 (11.34)	10 (12.50)	23 (10.90)	0.01
No	196 (67.35)	43 (53.75)	153 (72.51)	
Unknown	50 (17.18)	22 (27.50)	28 (13.27)	

*P*‐values for the variables age, time since diagnosis to data collection, and median time to death from diagnosis were calculated with the median test for differences in location. All other analyses were performed using Pearson's chi‐square, unless the expected cell count was <5, then Fisher's exact test was performed.

a High‐risk cytogenetics include FISH t(4;14), t(14;16), t(14;20), del(17/17p), gain(1q), nonhyperdiploid karyotype, karyotype del(13).^32^

Deep vein thrombosis (DVT) was the most common type of VTE experienced by our patients (64%), with 15% of patients experiencing a PE and 21% of patients experiencing multiple VTEs. The median time from MM diagnosis to VTE was 251 days (Q1 122, Q3 891), with 41% of patients experiencing a VTE more than 1 year after diagnosis. The most common single treatment phase for VTE was during induction therapy (30%), but the majority (70%) of VTEs were experienced after induction therapy and occurred throughout the disease course, including during maintenance (28%) and at relapse (11%). The least number of VTEs occurred on bortezomib containing regimens when used without an immunomodulator (13%) and for single‐agent immunomodulator therapy (6%) with an increased rate of VTE for immunomodulator combination regimens (56%), including with bortezomib (21%). There were 36 cases (45% of total VTE cases) who received an autologous stem cell transplantation (ASCT) in their disease course. Of those, 10 (28%) experienced a VTE within the peri‐transplant period (−30 days to +90 days from ASCT date).

Most patients in our study (82%) had more than one risk factor for VTE and thus were considered high risk for VTE according to the IMWG guidelines. We did not find an association between the baseline risk category, baseline prophylactic medication, and the interaction of baseline risk with baseline medication with the rate of future VTE (*P* = 0.16, *P* = 0.37, and *P* = 0.67, respectively). Neither baseline risk nor medications prescribed at baseline were able to predict the probability of experiencing a future clot. Moreover, there was no temporal relationship between risk and VTE. Specifically, high‐risk patients were not more likely to clot earlier than low‐risk patients (*P* = 0.45). During the course of treatment, six (7.5%) case patients with a low risk of VTE acquired additional risk factors for VTE and thus were then considered high risk at their time of clot. One of the six patients whose risk category increased had a change in thromboprophylaxis to warfarin or LMWH from aspirin, while five patients had no change from their baseline thromboprophylaxis (no drug n = 3 or ASA n = 2). The risk category at the time of VTE was found to be a better predictor of clot (*P* = 0.09) than baseline risk (*P* = 0.19) when the matching was controlled.

According to the IMWG guidelines, high‐risk patients should be given warfarin or LMWH and low‐risk patients should be given ASA. Of people prescribed warfarin or LMWH in our cohort, 100% were high risk (Table 3). However, 204 (85%) high‐risk patients were not on warfarin or LMWH. A total of 56 patients (19%) in our study (22 low‐risk and 34 high‐risk) were appropriately prophylaxed according to the IMWG guidelines. Only 14 (18%) of our case patients were on appropriate VTE prophylaxis. Among controls, 169 (80%) were considered high risk, with only 25 (15%) of these high‐risk controls receiving warfarin or LMWH per the guidelines. There were 34 high‐risk patients (14%, nine cases and 25 controls) treated appropriately according to guidelines. When we account for matching and only look at high‐risk patients, guidelines being followed and preventing a VTE are not associated with one another (*P* = 0.72).

**Table 3:** Baseline thromboprophylaxis type for cases and controls by number of IMWG risk factors

N (%)	No thromboprophylaxis (N = 94)			Aspirin use (N = 163)			LMWH or warfarin (N = 34)			Total cohort (N = 291)	
Risk Level	Case (N = 28)	Control (N = 66)	*P* ^A^‐value	Case (N = 43)	Control (N = 120)	*P* ^A^‐value	Case (N = 9)	Control (N = 25)	*P* ^A^‐value	*P* ^B^‐Value	*P* ^C^‐Value
Low (N = 53)	6 (21.4)	25 (37.9)	0.12	5 (11.6)	17 (14.2)	0.68	0 (0.0)	0 (0.0)	NA^a^	0.16	0.37
High (N = 238)	22 (78.6)	41 (62.1)		38 (88.37)	103 (85.8)		9 (100.0)	25 (100.0)			

*P* ^A^‐values compare differences in category of risk factors (low or high) and clotting (case vs control) for patients within each treatment category (no thromboprophylaxis, ASA, and LMWH/warfarin) using Pearson's chi‐square test.

a It is not possible to calculate a *P* ^A^‐value for LMWH or warfarin due to the multiple empty cells. *P* ^B^‐value evaluates an association between number of risk factors and clotting for the entire cohort (N = 291) when controlling for baseline thromboprophylaxis type. *P* ^C^‐value evaluates an association between baseline thromboprophylaxis type and clotting for the entire cohort (N = 291) when controlling for baseline risk category.

In addition to the IMWG risk factors, we performed an exploratory conditional logistic regression of other potential VTE risk factors. We were unable to identify any other risk factors that increased likelihood of clotting. We also explored two‐way interactions of risk factors using backwards and stepwise regression, but did not find any significant combinations of predictors in modeling the probability of experiencing DVT.

## 4 DISCUSSION

The purpose of this study was to investigate whether or not the IMWG guidelines for thromboprophylaxis in MM patients are being followed, and if so, determine whether the guidelines are effectively preventing future VTE. The IMWG model is intended for use in patients on IMID therapy, but we did not limit the model to this. Because we selected all qualifying cases from HERON and then matched them in an approximately 1:3 (cases:controls) manner, these descriptive results were not a random sampling of all MM patients at KUMC. Despite this, we had good matching of cases and controls overall, with the only significant difference being baseline Karnofsky performance status. We opted for this approach (ie, a case‐control study) for this initial investigation since VTEs are a rare event, and performed our statistical analysis accordingly. Because we oversampled cases relative to the general MM population with this design, our patients may appear to be underutilizing warfarin and/or LWMH, which may be confounding our results. Data would suggest that ASA, warfarin, and LMWH can reduce the risk of VTE in patients with MM^23^, ^27^; however, the majority of studies have been done in a relatively healthy, select group of patients who have been followed for 1‐2 years. KUMC is a tertiary referral center, and our dataset includes real‐world data. The worse Karnofsky performance score among our cases suggests our data may represent a more chronically ill population, though we could find no combination of risk factors that predicts specifically for VTE. Our median time to clot (251 days) agreed with prior publications that the majority of VTEs in MM occur within the first year; however, our data suggest that patients maintain a greater risk for VTE more than 1 year after diagnosis and throughout the treatment course than has previously been appreciated.^28^, ^29^, ^30^

The majority of our patients had more than 1 risk factor at the time of their MM diagnosis (82%) and were therefore considered high risk for VTE. Risk category at baseline, nor VTE prophylaxis medications, nor the interaction of risk with VTE prophylaxis was able to predict future cases of VTE. Our study found poor concordance with guidelines regarding ASA or LMWH use for VTE prophylaxis among patients with MM. Therefore, we cannot make conclusions on whether or not the IMWG guidelines are effective at preventing VTE. We also explored other potential risk factors in addition to the IMWG risk factors that may influence this prolonged risk of clotting in multiple myeloma (Table 1), but no other single or combined risk factors contributed significantly to having a future clotting event.

Risk category at the time of clot had a stronger association than baseline risk with the probability of clotting, suggesting that patients’ risk factors change over time. We observed six low‐risk cases who gained risk factors from the time of MM diagnosis to their time of clot, and thus were considered high risk at the time of their clot. However, only one of those cases had appropriate VTE prophylaxis changes associated with their increased risk. This data suggest that risk factors for VTE can change over the disease course, and, at least in our study, the heightened risk for VTE does not result in corresponding changes in VTE prophylaxis. This fluid risk profile indicates that MM patients require continual assessment of VTE risk and thromboprophylaxis monitoring throughout their disease course. Our study also highlights a concern for patient compliance among a general MM population. The concern for patient compliance raised in our study carries further implications among patients with MM given the widespread use of oral chemotherapeutics for disease treatment and suggests that further study of patient compliance is warranted. Our study was conducted prior to the widespread use of direct‐acting oral anticoagulants (DOACs). DOACs provide a significant advantage for ease of use without needs for continual blood monitoring, but remain expensive and not universally covered for VTE prophylaxis in malignancy. As data are emerging on the efficacy of DOACs in cancer patients,^31^ the opportunity for improved patient compliance should be included in analysis. Physician compliance is also of concern in the treatment of MM given the high rate of patients not reported to be on any VTE prophylaxis in our study. The IMWG guidelines contain numerous risk factors that must be considered when determining the risk of VTE for any given patient, and can be cumbersome when used in clinical practice. Further identification of the explicit risk factors for VTE in MM may lead to a simplified algorithm with higher rates of implementation.

Our single institution case‐control study design minimized the potential for confounding by matching cases to controls by age, gender, and time of diagnosis, which was strength of our study. The availability of electronic medical records (EMR) dating back to 2007 allowed for easier and more accurate collection of patient data. The EMR also allowed us to adequately capture the prescribing of warfarin and LMWH. Alternatively, ASA can be purchased over the counter without a prescription and usage is harder to track via an electronic medical record, possibly accounting for the 34% of patients taking no medication for thromboprophylaxis. Our retrospective design limited our ability to identify mitigating factors that may have limited the usage of LMWH or warfarin. Incomplete charting within the medical record made it difficult to capture complete risk factor data on all patients and limited our sample size and analyses. As with all studies, excluding missing data introduces the opportunities for selection bias if missing data fields was systematically associated with other key study measures, but the overall survival between groups was similar (*P* = 0.35), and thus, our concerns over such biases were attenuated. While the data exclusion may have limited the study power, this work fills an important gap by providing initial effect sizes that could be utilized to better assess power for subsequent, confirmatory research on this topic. There are multiple possible reasons for poor compliance of the IMWG recommendations for VTE prophylaxis including a lack of clinician education, patient reluctance or inability to take recommended medications, and issues with cost or monitoring needed for anticoagulation that cannot be appreciated through simple chart extraction. The lower Karnofsky performance status in our case population compared to controls may highlight a population of patients in whom anticoagulation was contraindicated or challenged. We did not collect data on monitoring warfarin levels, and therefore, we cannot know the level of compliance in our population.

With a median follow up of 5 years, our study does not demonstrate an increased risk of death for patients experiencing VTE. Our study did not analyze other long‐term impacts of VTE among our patients, but is warranted given the high rates of VTE. Further follow up is needed to ultimately determine the impact of VTE on patient mortality in our study. Additionally, our study did not look at the rates of complications from VTE prophylaxis and the impact of side effects on usage patterns is not known. Ultimately, our study demonstrates that VTE remains a significant issue for patients with MM, and further study is warranted to best identify and prevent VTE.

Overall, the purpose of this study was to investigate the efficacy of the IMWG guidelines and determine whether additional risk factors could improve accuracy of identifying patients who will likely clot. The observed poor concordance with IMWG guidelines indicates a gap in clinical practice and offers an opportunity to improve physician awareness and implementation of the guidelines. While we did not identify any additional risk factors, we did discover that patients require continual risk assessment throughout their disease course, rather than just at baseline. Given the potential design influences, it is important to replicate this finding through further studies, but ultimately, our study suggests important gaps in clinical practice that can be addressed by clinicians to improve outcomes in patients with multiple myeloma.

## CONFLICT OF INTEREST

None declared.
